# Migrasomes: From Biogenesis, Release, Uptake, Rupture to Homeostasis and Diseases

**DOI:** 10.1155/2022/4525778

**Published:** 2022-04-14

**Authors:** Yaxing Zhang, Wenhai Guo, Mingmin Bi, Wei Liu, Lequan Zhou, Haimei Liu, Fuman Yan, Li Guan, Jiongshan Zhang, Jinwen Xu

**Affiliations:** ^1^Department of Physiology, School of Basic Medical Sciences, Guangzhou University of Chinese Medicine, Guangzhou, Guangdong 510006, China; ^2^Research Center for Integrative Medicine (Key Laboratory of Chinese Medicine Pathogenesis and Therapy Research), Guangzhou University of Chinese Medicine, Guangzhou, Guangdong 510006, China; ^3^Department of Traditional Chinese Medicine, The Third Affiliated Hospital, Sun Yat-sen University, Guangzhou, Guangdong 510630, China; ^4^Institute of Integrated Traditional Chinese and Western Medicine, Sun Yat-sen University, Guangzhou, Guangdong 510630, China; ^5^Graduate School of Guangzhou University of Chinese Medicine, Guangzhou, Guangdong 510006, China; ^6^Department of Otorhinolaryngology, The Seventh Affiliated Hospital, Sun Yat-sen University, Shenzhen, Guangdong 518107, China

## Abstract

Migrasomes are migration-dependent membrane-bound vesicular structures that contain cellular contents and small vesicles. Migrasomes grow on the tips or intersections of the retraction fibers after cells migrate away. The process of releasing migrasomes into the extracellular space is named as “migracytosis”. After releasing, they can be taken up by the surrounding cells, or rupture and further release their contents into the extracellular environment. Physiologically, migrasomes provide regional cues for organ morphogenesis during zebrafish gastrulation and discard the damaged mitochondria in response to mild mitochondrial stresses. Pathologically, migrasomes are released from podocyte during early podocyte stress and/or damage, from platelets after infection with severe acute respiratory syndrome coronavirus 2 (SARS-CoV-2), from microglia/macrophages of the ischemic brain, and from tumor necrosis factor *α* (TNF*α*)-activated endothelial cells (ECs); thus, this newly discovered extracellular vesicle is involved in all these pathological processes. Moreover, migrasomes can modulate the proliferation of cancer cell *via* lateral transferring mRNA and protein. In this review, we will summarize the biogenesis, release, uptake, and rupture of migrasomes and discuss its biological roles in development, redox signalling, innate immunity and COVID-19, cardio-cerebrovascular diseases, renal diseases, and cancer biology, all of these highlight the importance of migrasomes in modulating body homeostasis and diseases.

## 1. Introduction

During the process of cell migration, large vesicles grow on the tips or at the intersections of retraction fibers; eventually, the retraction fibers break, and the pomegranate-like structures (PLSs, referred as “migrasomes”), within a single-limiting membrane, are released into the extracellular space from retraction fibers at the rear of migrating cells; the released PLSs can be directly taken up by the surrounding cells [1, 2]; or they will rupture/disappear and release their contents into the environment in a migration-dependent release process named “migracytosis” [1–4]. The primary function of PLSs is intercellular communication, similar to the mechanisms such as exocytosis and exosome release [1, 2, 5]. This novel extracellular vesicle (EV) has displayed many biological functions. Migrasomes provide regional cues for organ morphogenesis during zebrafish gastrulation [3]. Damaged mitochondria can be discarded *via* migrasomes, which highlight the essential role of migrasomes in modulating mitochondrial quality control process and redox signalling [6]. Severe acute respiratory syndrome coronavirus 2 (SARS-CoV-2) induces platelets to release migrasomes and initiates programmed cell death of platelets in severe coronavirus disease 2019 (COVID-19) [7]. Moreover, tumor necrosis factor *α* (TNF*α*) induces the formation of migrasomes in human coronary artery endothelial cells (ECs) [8], parenchymal migrasomes were formed during ischemic brain injury [9], the podocyte-released migrasomes in urine are indicators for early podocyte injury [10], and migrasomes can also modulate cancer cell proliferation *via* lateral transferring mRNA and protein *in vitro* [2]. In this review, we will summarize the current understanding of migrasomes from biogenesis, release, uptake, and rupture to its roles in homeostasis and diseases; and discuss the perspective of migrasomes based on the keywords “migrasome” or “migrasomes” according to the official publishing in PubMed and Web of Science before January 22, 2022.

## 2. The Discovery of Migrasomes/Migracytosis

The long projections from the surface of cells or the retraction fibrils/fibers from the migrating cells have been observed by Porter [11] and by Taylor [12], respectively; however, retraction fibers have received little attention despite their widespread presence in different cell types [1]. Dr. Yu found “a structure closely resembling an opened pomegranate stood outside a cell” by transmission electron microscopy; the particular image was unusual because there were several of these large structures outside the cell: some of them were empty, some of them had a few vesicles inside, and some of them were packed with vesicles [1, 13]. In 2015, the milestone about PLSs was published in *Cell Research*; Yu's team isolated PLSs by subcellular fractionation *via* density gradient centrifugation and confirmed by transmission electron microscopy [1]. They identified PLS proteins by mass spectrometry analysis and identified tetraspanin-4 (TSPAN4) as the clearest PLS marker by using green fluorescent protein (GFP)-tagged candidate proteins [1]. Besides TSPAN4, wheat-germ agglutinin (WGA), a lectin that binds specifically to sialic acid and N-acetyl-d-glucosamine [14], can also label migrasomes in living cells [15]. Time-lapse imaging revealed that the average lifespan of a PLS derived from normal rat kidney (NRK) cell is about 200-400 min [1, 4]. Using the inhibitor or promoter of migration, they confirmed that the formation of PLSs is migration-dependent (see detail in “The current understanding of migrasomes biogenesis”) [1]. Migrasomes are not an artifact caused by culturing cells on a highly rigid surface such as glass or plastic; these PLSs were widely existed in many tissues and cells *in vivo* and *in vitro* [1]. By overexpressing GFP as a tracer for the cytosolic contents in TSPAN4-mCherry-expressing NRK cells, they discovered that the cytosolic components/material and vesicles of unknown origin can actively enter into migrasomes; then, migrasomes and their contents are released into the extracellular space [1, 4]. To their astonishment, they observed that migrasomes left by one cell can be taken up by the surrounding cells [1, 2]. These indicated that migrasomes might play essential roles in modulating intercellular communication.

## 3. The Basic Features of Migrasomes

The detail methods for visualizing migrasomes by fluorescence microscopy and electron microscopy have been well established by Dr. Chen in Yu's lab [16]. As the novel vesicle structure, we have also summarized the basic differences between migrasomes and exosomes in June, 2020 [17]. The number of smaller vesicles in migrasomes varies greatly; some migrasomes contain up to 300 vesicles, while most contain fewer than 10 [1]. What are these smaller vesicles? The diameters of exosomes are 30-200 nm, which are smaller than that of migrasomes (0.5-3 *μ*m in NRK cells and 1.87 ± 0.18 *μ*m in mice brain) [17]. However, it is unclear whether these smaller vesicles in migrasomes contain exosomes and whether the exosomes reported previously in the literature are partially released by migrasomes [1]. The protein types in migrasomes are not always the same, as that these in NRK cells and in the infarcted brain parenchyma of mice have been shown to be different [1, 9]. Although migrasomes from infarcted brain parenchyma of mice or NRK cells contain RNA-binding proteins, co-staining of F4/80 and RNA did not show RNA signalling within the migrasome either in cerebral cortex or white matter tracts [1, 9]. In contrast, Yu et al. found mRNA in migrasomes from L929 cells (the mouse fibrosarcoma cell line [18]) [2]. Liu et al. have found that miRNA exist in human podocyte cell lines (HPCs)-derived migrasomes, and its miRNA expression profile is different from these in exosomes derived from HPCs [10]. In addition, Antje et al. had identified DNA-interacting proteins in migrasomes from the infarcted brain parenchyma of mice; however, they have not investigated the DNA signalling in these migrasomes [9], and whether the migrasomes of a certain type of cell in a particular state contain DNA is unclear.

**Figure 1:**
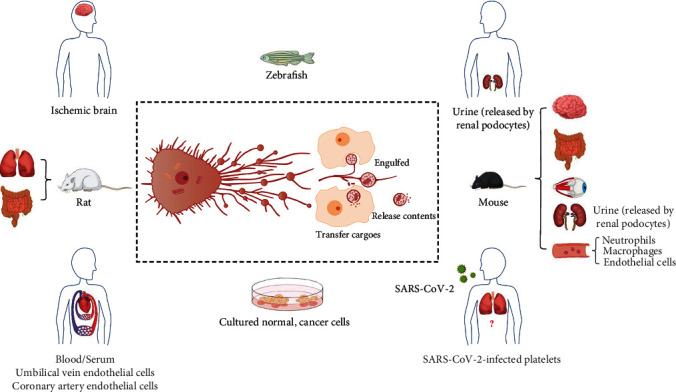
The formation, distribution, and function of migrasomes. Migrasomes are formed and released from the retraction fibers during cell migration; these migration-dependent membrane-bound vesicular structures contain many small vesicles, miRNA, mRNA, proteins, and the swollen mitochondria (the red ones in cell and migrasomes). After releasing, they can be engulfed by the surrounding cells, and transfer their cargoes into the surrounding cells, or rupture and further release their contents into the extracellular environment. Migrasomes are extensively distributed in many cultured cells *in vitro*, such as cancer cells [L929 (mouse fibrosarcoma cell), MDA-MB-231 (human breast cancer), SKOV-3 (human ovarian adenocarcinoma), HCT116 (human colon cancer), SW480 (human adenocarcinoma), MGC803 (human gastric carcinoma), MIACaPa-2 (human pancreatic cancer), and B16 (mouse melanoma)]; the normal human cells [HaCaT (human keratinocyte) and HPC (human podocyte cell)]; the normal mouse cells [primary macrophages, neuron, and embryonic stem cells, RAW 264.7 (mouse monocyte-macrophage), MEF (mouse embryonic fibroblast), and NIH3T3 (mouse embryonic fibroblast)]; and normal rat kidney (NRK) cell. Migrasomes are also found in the key organs of human, mouse and rat *in vivo*. Physiologically, migrasomes serve as chemoattractants to affect organ morphogenesis in zebrafish *in vivo*. Mitocytosis is required for maintaining mitochondrial membrane potential (MMP) and viability in neutrophils in mouse *in vivo*. Migrasomes formed in carbonyl cyanide 3 chlorophenylhydrazone (CCCP)-treated human umbilical vein endothelial cells (ECs) *in vitro* also contribute to maintaining mitochondrial homeostasis. Tumor necrosis factor *α* (TNF*α*) induces the formation of migrasomes involved in cell-cell signalization between migrating primary human coronary artery ECs. Pathologically, migrasomes transfer mRNA and protein to modulate the proliferation of cancer cell *in vitro*. Podocyte-released migrasomes in urine serve as an indicator for early podocyte injury in mouse and in human *in vitro*. Migrasomes are in the ischemic brain of mouse and human in vivo, and are involved in the pathological process of ischemic stroke. Migrasomes are detected in human serum samples, and they are released from the platelets in human infected with severe acute respiratory syndrome coronavirus 2 (SARS-CoV-2). Migrasomes are also found in rat lung and intestine; in mouse intestine and eye, they tend to be located inside cavity, such as blood vessel or pulmonary alveoli; however, their roles in lung injury of severe coronavirus disease 2019 (COVID-19) still need further investigation.

Migrasomes extensively distribute in normal human, mouse or rat cells *in vitro*, in cancer cells *in vitro*, and in human, mouse, rat, and zebrafish *in vivo* [1, 3, 6, 9, 10, 19–21] (Figure 1). For example, the differentiation of osteoclasts from murine monocyte-macrophage cell line (RAW 264.7 cells) stimulated by receptor activator of nuclear factor *κ*-B ligand (RANKL) can induce the formation of vesicle resemble “migrasomes” [21]. Therefore, the location of these PLSs seems to confer them with different biological functions and to investigate their expression patterns in different cells or tissues are of great importance.

## 4. The Current Understanding of Migrasome Biogenesis

Cell migration is a physically integrated multistep process [22, 23]. To migrate, a cell must acquire a spatial asymmetry enabling it to turn intracellularly generated forces into net cell body translocation; one manifestation of this asymmetry is morphological polarization, i.e., a clear distinction between cell front and rear [22]. An important consequence of polarization is that extension of active membrane processes, which takes place primarily around the cell front [22]. Therefore, for migration to occur, extend protrusions in the direction of migration must form and then stabilize by attaching to the surroundings [23]. These protrusions can be broad, flat, sheet-like structures, named “lamellipodia,” or thin, cylindrical, needle-like projections, named “filopodia” et al.; they are usually driven by actin polymerization, by the cortical expansion mechanism, the Brownian ratchet mechanism, or a combination of these [22, 24]. These protrusions are stabilized by adhering to extracellular matrix (ECM) or adjacent cells *via* the transmembrane migration-promoting receptors, which, as the “feet” of a migrating cell, linked to the actin cytoskeleton *via* adaptors [22, 23]. These adhesions serve as traction sites for migration as the cell moves forward over them [23]. Once the protrusions have become adherent to the substratum, translocation of the cell body forward may occur by myosin interactions with actin filaments, possibly *via* relative movement of adhesion complexes across cortical actin filament “tracks,” or the contraction of filaments connecting cell-substratum adhesion complexes with intracellular structures; in either case, the magnitude of traction is greater than the rearward pull on the adhesion complexes [22]. At the rear of the cell, the adhesions are released as the trailing edge detaches from the substratum (here, the magnitude of traction is less than the contraction force), thus allowing net translocation of the cell in the direction of movement and completing a migratory cycle [22, 25]. Therefore, the polarity is intrinsic to a migrating cell, and the basic cell migration cycle includes extension of a protrusion from cell membrane in the direction of movement, formation of stable attachments near the leading edge of the protrusion, translocation of the cell body forward, release of adhesions, and retraction at the cell rear [22, 23, 25–30].

Time-lapse imaging revealed that formation of migrasomes is likely related to cell migration [1]. Therefore, if migrasome is dependent on migration, modulation events/process involved in migration will influence the formation of migrasome. Indeed, the number or formation of PLSs was largely reduced when reducing cell migration speed by migration inhibitors [1]: the myosin II inhibitor “blebbistatin” [31, 32], and a cell-permeable dynamin inhibitor “dynasore” [33], which has been shown to suppress lamellipodia formation and cancer cell invasion by destabilizing actin filaments [34]. As we have mentioned above, lamellipodia or filopodia formation is usually driven by actin polymerization. The polymerized actin fibers are closely associated with the membrane of some migrasomes [1]. So, what is the function of actin polymerization in migrasomses? Suppressing actin polymerization with cytochalasin B or latrunculin A, or blocking formation of branched actin networks with CK636 (an inhibitor of the Arp2/3 complex, and Arp2/3 complex is an important actin filament nucleator that creates branched actin filament networks required for formation of lamellipodia and endocytic actin structures [35]), reduces the number of PLSs by preventing forming new migrasomes [1]. Thus, actin polymerization is likely required for migrasome formation, either by affecting cell migration as some of these actin polymerization inhibitors can also inhibit migration or by directly involving in migrasome biogenesis [1]. In contrast, the number of migrasomes was increased when accelerating cell migration by different strategies (see detail in below) [1, 4]. Recently, Lu et al. have identified 507 compounds which had significant inhibitory effect on migrasome generation, and 463 out of these 507 hits showed no or less retraction fibers indicating defect of cell migration; this further confirmed that generating migrasome is dependent on migration [36]. Based on migration-dependent biogenesis of PLSs, these PLSs were named as “migrasomes” [1]. Hu et al. has identified “accessible cholesterol”-rich particles released from the macrophage; they are about 30 nm and represent fragments of the plasma membrane that are pulled away and left behind during the projection and retraction of filopodia and lamellipodia [37]. This particle release was abolished when the movement of filopodia/lamellipodia was blocked by blebbistatin or by actin depolymerization (latrunculin A), and their release was increased if the disassembly of focal adhesions (the macromolecular complexes that tether cells to the underlying substrate) was suppressed by FAK inhibitor (CAS 4506-66-5) [37]. Thus, future study focusing on the biological characteristics of these particles and migrasomes is required.

The functional units of cell adhesion typically include cell adhesion molecules/adhesion receptors, the ECM proteins, and the cytoplasmic plaque/peripheral membrane proteins [24]. Among these above, cell adhesion receptors, including members of the integrin, cadherin, immunoglobulin, selectin, and proteoglycan (e.g., syndecans) superfamilies, are usually transmembrane glycoproteins that mediate binding interactions at the extracellular surface and determine the specificity of cell-cell and cell-ECM recognition [24]. Integrins are *αβ* heterodimers with a large extracellular domain that binds the ECM and links to the actin cytoskeleton by a short cytoplasmic tail [38]. The extracellular domain of integrins determines the binding specificity and recognizes diverse matrix ligands including fibronectin (e.g., *α*5*β*1, *α*v*β*3, and *α*4*β*1), collagen (e.g., *α*1*β*1 and *α*2*β*1), and laminin (e.g., *α*2*β*1, *α*3*β*1, and *α*6*β*1) [38]. Both integrins and TSPAN4 are highly enriched on migrasomes; unlike TSPAN4 that is also abundant on retraction fibers, integrins are only present at very low levels on retraction fibers [1, 4]. Moreover, TSPAN4 was on the upper side, while the endogenous integrin *α*5 and *β*1 were enriched on the bottom of migrasomes, and Yu has confirmed that the integrin-enriched regions on migrasomes are not focal adhesions (FAs) [4]. The number of PLSs was increased when accelerating cell migration by knocking down SHARPIN (an endogenous inhibitor of *β*1-integrin activation), and fibronectin increased migrasomes number per cell in a dose-dependent manner [1, 36, 39]. GLPG0187, the inhibitor of integrin *α*5*β*1, inhibited the biogenesis of migrasomes in a concentration-dependent manner without cytotoxicity [36]. The integrin *α*5 mRNA levels are much higher than other examined integrins (*α*1, *α*2, *α*3, and *α*6) in TSPAN4-GFP-expressing NRK (NRK-TSPAN4-GFP) cells [4]. Therefore, these cells produced more migrasomes on cover glasses coated with *α*5-pairing fibronectin than with other integrin-pairing laminin 511 or collagen I, and very few migrasomes formed on noncoated cover glasses [4]. Moreover, knockdown of ITGA5 that encodes *α*5 impaired the formation of migrasomes on cells cultured on fibronectin, but not on other ECMs [4]. Overexpressed integrin *α*1 or *α*3 in different cells enhanced migrasome formation, cell spreading, and migration on their corresponding ECM partner protein, but not on other ECM proteins [4]. Therefore, pairing of integrin with its specific ECM partner is a determinant for migrasome formation.

Recently, using chemical screening and RNAi, Yu' team identifies ROCK1 (a positive regulator of microfilament bundle and focal adhesion assembly [40]), rather than ROCK2, as a regulator of migrasome formation [36]. ROCK1 contributes to the formation of migrasomes *via* its role in adhesion to fibronectin to generate a traction force [36]. The role of adhesion on migrasome formation has also been confirmed *in vivo*. The *itgb1b*^*−*/*−*^ (encode integrin *β*1b) zebrafish embryos formed significantly fewer migrasomes at the gastrulation stages without impaired the speed of cell migration during gastrulation, which implies that integrin *β*1b in zebrafish gastrulas most probably regulates migrasome formation by providing adhesion [3]. Saito et al. have evaluated the potential of peptide scaffolds on the forming of migrasomes in cell culture; they found that the peptide interface comprising cell-penetrating peptides (pVEC and R9) and virus fusion peptide (SIV) have superior properties for enabling cell migration and migrasome formation than fibronectin protein, integrin-binding peptide (RGD), or bare substrate [20]; and these will help us to establish cell model for investigating migrasomes.

The regulatory processes of migrasome biogenesis discussed above were investigated based on migration-dependent characteristics of PLSs. TSPAN4 has been shown as a clearest PLSs marker on migrasomes. However, what is the function of this protein? Indeed, TSPAN4 is a key mediator for migrasome formation [41]: overexpression of 14 (1, 2, 3, 4, 5, 6, 7, 9, 13, 18, 25, 26, 27, and 28) out of the 33 known mammalian TSPANs in NRK cells enhanced migrasome formation, and among these 14 TSPANs, 9 TSPANs (1, 2, 4, 6, 7, 9, 18, 27, and 28) had a strong effect [41]. On the contrary, TSPAN4 deficiency impairs migrasome formation in NRK cells and MGC-803 cells, while knockout of TSPAN4 did not impair migrasome formation in L929 cells [41]. It seems that migrasomes-forming TSPANs have the compensative effect for the loss of TSPAN4, or TSPAN4 totally has no function in migrasome formation in L929 cells. Dynamically, TSPAN4 is recruited to the migrasomes from the retraction fibers during the migrasomal growth phase; in terms of organization, TSPAN4 forms discrete fast-moving puncta that concentrate on the migrasomal surface; and TSPAN4 cannot move from the migrasomes to the retraction fibers once it is recruited to the migrasomes [41]; however, the mechanisms of TSPAN4 nonreturn are not clear. TSPAN4 in migrasomes is about 4 times higher than in retraction fibers [41]. It is easy to think that TSPAN4 alone cannot form a migrasome. The proteins that interact with TSPAN4 might be another breakthrough to answer this question. TSPAN4 belongs to TSPANs family, which includes 33 members in human beings [17]. It has been well known that TSPANs, combined with a set of TSPANs-associated proteins and a high concentration of cholesterol, form a functional unit in cell plasma membranes, named TSPAN-enriched microdomains (TEMs) [17, 41, 42]. Migrasomes were also enriched with other TEMs components, such as integrins and other TSPANs, e.g., TSPAN1, TSPAN2, TSPAN27/CD82, TSPAN28/CD81, and cholesterol, which is about 40-fold in migrasomes relative to retraction fibers [4, 41]. Besides TSPANs and integrins, TEMs component cholesterol is also necessary for migrasome formation as that its formation was impaired when reducing cellular cholesterol levels [41]. The migrasomal membranes were several microns in size, while the typical TEMs are around 100 nm; therefore, the migrasomal membrane is a “TSPANs- and cholesterol-enriched macrodomains (TEMAs)” [41]. Yu's team has established a modified version of the *in vitro* migrasome formation system using the artificially generated giant unilamellar vesicles (GUVs) *via* electrofusion of the proteoliposomes and manually pulling the GUVs membrane by a glass needle; they showed that the biogenesis of migrasomes is mediated by assembling the 100-nm scale TEMs, which exist in the tether membrane, into the micron-scale macrodomains as “TEMAs,” and then, these TEMAs swell into the large vesicle-like migrasomal shape [41].

Therefore, TSPANs, cholesterol molecules, and integrins are necessary components for mediating migrasome biogenesis [3, 4, 41]. Mechanistically, active integrins are assembled into puncta on retraction fibers prior to migrasome formation, and the interactions of correct pairing of integrin complexes with its specific ECM partner protein establish the adhesion sites along the retraction fiber, which then serve as platforms for migrasome formation [4, 13]. The integrins on the cell body enable the cell to migrate, whereas the integrins on the migrasomes provide the adhesion for retraction fiber tethering [3, 4]. The mechanical stress exerted along the retraction fibers at the rear of the migrating cell induces the formation of TEMAs *via* triggering the cluster of TSPANs (e.g., TSPAN4 and TSPAN7) and cholesterol molecules; TEMAs enrichment causes the stiffening of the plasma membrane, thus facilitating a new migrasome formation [3, 41, 43].

## 5. The Current Understanding of the Biological Functions of Migrasomes

### 5.1. Migrasomes Coordinate Organ Morphogenesis *via* Serving as Chemoattractants

Migrasomes exist *in vivo*, what are their physiological functions in living organisms? The robust movement of cells during gastrulation of zebrafish embryo, the optical clarity, and out-of-mother development facilitating high-quality imaging make zebrafish embryo as a promising model to visualize, investigate, and characterize endogenous extracellular vesicles (EVs) in real-time and expand our understanding of EVs biology at cellular and systems level [3, 44–47]. The embryonic cells during zebrafish gastrulation generate long projections and migrasomes, which are present in the pockets between the blastodermal margin and the yolk syncytial layer, and in the extracellular pockets of the space between mesendodermal cells [3]. By developing *itgb1b*^*−*/*−*^ embryos, maternal zygotic (MZ) tspan7- and MZtspan4a-mutant embryos, TSPAN7 and 4a, and integrin *β*1b have been confirmed as the key molecules mediating the formation of migrasomes in zebrafish gastrulas [3]. Physiologically, migrasomes act as a source of Cxcl12 during zebrafish gastrulation, and migrasomes are enriched on a large cavity underneath the embryonic shield where they serve as chemoattractants through delivering Cxcl12a for Cxcl12a–Cxcr4b signalling axis to ensure the correct positioning of dorsal forerunner cells vegetally next to the embryonic shield, thereby affecting organ morphogenesis [3].

In addition to zebrafish, migrasome-like structures are also in mouse embryonic stem cells and embryonic fibroblast *in vitro* [1] (Figure 1), while the function of migrasomes in mouse embryonic development has not been identified. Mammalian fertilization comprises sperm migration via the female reproductive tract, biochemical and morphological changes to sperm, and sperm-egg interaction in the oviduct [48]. Motility is one of the most remarkable characteristics of mammalian spermatozoa, while it is not clear whether sperm can produce or release migrasomes; if can, what are the functions of migrasomes in mammalian fertilization?

### 5.2. Migrasomes Discard the Damaged Mitochondria: An Essential Role in Oxidative Stress

On 2021 May 27, a study published in *Cell* by Yu's lab has showed that migrasomes contain multiple mitochondria [6]. They found that the oxidative phosphorylation uncoupler carbonyl cyanide 3 chlorophenylhydrazone (CCCP) induces loss of mitochondrial membrane potential (MMP), and generation of high reactive oxygen species (ROS) in mitochondria; subsequently, these damaged mitochondria with an average size of 240 nm and swollen cristae signal their status and subsequently move to the cell periphery through their intrinsic avoidance of binding the inward motor protein dynein, and through globally enhancing the recruitment of KIF5B (the outward motor protein kinesin superfamily protein 5B [49]) to mitochondria; thus, KIF5B selectively binds damaged mitochondria and pulls them to plasma membrane, where myo-19 (myosin-19, an actin-based outer mitochondrial membrane motor, such as propelling mitochondria to filopodia tips [50–52]) tethers mitochondria to cortical actin, which is tightly associated with the plasma membrane [6]. The tips of tubular mitochondria bind to cortical actin and undergo Drp1 (the mitochondrial fission factor [53])-mediated fission, and then, they are sent into migrasomes; these migrasomes containing damaged mitochondrion were referred as “mitosomes”; finally, damaged mitochondria with deleterious mtDNA mutations are selectively disposed of by migrasomes release, a process named as “mitocytosis” [6].

By genetically manipulating the expression of TSPAN4, TSPAN9, dynein, KIF5B and integrin, and by blocking migration using the myosin II inhibitor “blebbistatin” in L929 cells, Jiao et al. found that blocking mitocytosis causes loss of MMP and reduction of spare respiration capacity in cells that are not exposed to mitochondrial stressors, while enhanced mitocytosis improves the loss of MMP and preserves the spare respiration capacity in cells with or without CCCP treatment [6]. Besides CCCP, other mitochondrial stressors, such as deferiprone (a typical iron chelator [54, 55]), antimycin A (a complex III inhibitor [56]), and oligomycin (a selective F1FO-ATPase inhibitor [57, 58]), and starvation can also induce mitocytosis in L929 cells [6]. CCCP treatment also induces mitocytosis in mouse bone marrow-derived macrophages, human pancreatic cancer cells (MIACaPa-2 [59]), and human umbilical vein ECs [6]. Therefore, mitocytosis is a general mechanism that is activated by various mitochondrial stresses in a variety of cells [6].

### 5.3. Migrasomes in Innate Immunity and COVID-19: Still Long Way to Go

The classical EVs, including exosomes (30-200 nm), microvesicles (approximately 200 nm), and apoptotic bodies (1-2 *μ*m) [17, 60, 61], can convey pathogen molecules that serve as antigens or agonists of innate immune receptors to induce host defence and immunity, or that serve as regulators of host defence and mediators of immune evasion [60]. The functions of EVs on innate immunity are conferred partially by transferring pro- or anti-inflammatory mediators, membrane receptors, enzymes, mRNAs, and noncoding RNAs to the targeting cells and by the interaction of EVs with the complement and coagulation systems [61–65]. Migrasomes are newly discovered EVs and also contain proteins and nucleic acids; what are the functions of migrasomes in regulating innate immunity?

Two studies have shown that macrophages are capable of generating migrasomes [1, 6]. The bone marrow-derived macrophages (BMDMs) derived from TSPAN9^−/−^ mice have significant reduced migrasome numbers and loss of MMP compared to those from wild-type (WT) mice; re-expressing TSPAN9 in TSPAN9^−/−^ macrophages will partially restore the impaired migrasome formation and improve the loss of MMP in cells grown on the untreated migrasome-forming surface, rather than on a hydrophilic surface, which significantly reduces the migration and does not support migrasome formation [6]. These indicated that loss of membrane potential is possibly caused by reduced mitocytosis, rather than by a function of TSPAN9 independent of mitocytosis.

There are migrasomes in human blood, although their origin is not clear [19]. Jiao et al. found that migrasomes were extensively generated by circulating neutrophils in mice, as that around 87% of migrasomes from blood originated from neutrophils [6]. Some neutrophil-derived migrasomes can adhere to vessels in the circulation for a long time, while some are detached quickly after generation [66, 67]. Most importantly, the migrasomes generated by neutrophils contain damaged mitochondria [6]. Migrasome formation by neutrophils from TSPAN9^−/−^ mice is significantly reduced, and the percentage of spleen neutrophils with higher MMP is greatly reduced in TSPAN9^−/−^ mice when compared with the WT, while there is almost no difference in MMP between WT and TSPAN9^−/−^ bone marrow neutrophils, as that bone marrow neutrophils have not yet undergone their long-distance migration; therefore, migrasome/mitocytosis has not yet kicked in; thus, this confirmed that it is migrasome, but not TSPAN9, that contribute to mitochondrial quality control [6].

So, what is the consequence of controlling mitochondrial quality by mitocytosis in immune cells? Jiao et al. showed that the generation and maturation of neutrophils are normal in TSPAN9^−/−^ mice; in contrast, the number of neutrophils is reduced in the spleen from TSPAN9^−/−^ mice compared with WT [6]. When injecting TSPAN9^−/−^ or WT bone marrow neutrophils to WT mice, WT neutrophils significantly outnumber TSPAN9^−/−^ neutrophils after one day's circulation; thus, mitocytosis physiologically contributes to the viability of neutrophils in the circulation [6]. Therefore, disposal of damaged mitochondria *via* releasing migrasomes is essential for keeping circulating neutrophils alive; however, these are limited to the mice in the steady state [6, 67], and using the infectious animal models or patients to explore the functions of migrasomes in other migratory immune cell development and in antiviral innate immunity will be more excited.

With almost two years into the severe coronavirus disease 2019 (COVID-19) pandemic, the impacts of the severe acute respiratory syndrome coronavirus 2 (SARS-CoV-2) go far beyond the suffering and death caused by COVID-19 itself. Scientists around the world are actively investigating SARS-CoV-2 and looking for the effective prevention and control strategies both in modern medicine and in traditional Chinese medicine. COVID-19 is characterized by pneumonia, lymphopenia, exhausted lymphocytes, and a cytokine storm [68]. Moreover, many patients with COVID-19 present with hypercoagulation and thrombosis [69, 70]. In these patients, platelets become activated and aggregate; these hyperactive platelets activate monocytes, leading to monocyte tissue factor release and thus contributing to the overwhelming thromboinflammation [7, 70–73]. Koupenova et al. revealed that platelets internalize SARS-CoV-2 in an angiotensin-converting enzyme 2 (ACE2)-dependent manner, or in an ACE2-independent manner by attaching to platelet-derived microparticles, and viral internalization leads to rapid digestion, programmed cell death, and release of EVs from platelets, such as microparticles, exosomes, and migrasomes [7]. Rapid platelet death after viral uptake indicates that the platelet milieu does not permit viral replication; this may be protective in immune response; however, the release of platelet contents during dying can be highly prothrombotic or proinflammatory and can lead to dysregulated immune activation [7]. Therefore, these indicated that migrasomes might contribute to thromboinflammation in COVID-19.

Exosomes are also implicated in the pathogenesis of COVID-19; for example, SARS-CoV-2 RNA is present in the exosomal cargo, which suggests that the virus might use the endocytosis route to spread infection [74–81]. Similar to exosomes, migrasomes can also transport cargoes, such as proteins and nucleic acids [2]. Besides in the platelets, brain, blood, and urine of human; in blood, urine, brain, intestine, eye, neutrophils, and macrophages of mouse; and in lung and intestine of rat [1, 6, 7, 9, 10, 19], we do not know the detail distribution and function of migrasomes in human under physiological or pathological conditions. If migrasomes are extensively produced and/or distributed in human body, it seems that they might be more important in SARS-CoV-2 infection and in the pathogenesis of COVID-19. However, this is a bold scientific hypothesis, and it still needs to be carefully verified.

### 5.4. Migrasomes in Cardio-Cerebrovascular Diseases: Consequence or Contributor?

In the ultrathin sections of mouse or rat tissues, migrasomes tend to be present inside cavity such as blood vessel and pulmonary alveoli, for example, in ECs [1]. TNF*α* induces migrasome formation in ECs, and this formation is highly dependent on cell-cell and cell-ECM interaction, indicating that migrasomes play essential role in the transmission of F-actin-based mechanical forces for proper polarization of adjacent cells and coordination of the cell migration direction [8]. Tropomyosin-1 (a coiled-coil protein that wraps around the actin molecules and provides stability to actin filaments [82]) is a key regulator of TNF*α*-mediated migrasome formation in ECs, as that angiogenic capacity and migrasome formation were augmented if tropomyosin-1 was knockout in TNF*α*-activated ECs [8]. CCCP-mediated migrasome formation and mitocytosis in ECs might contribute to mitochondrial quality control process [6]. It has speculated that migrasomes might be a particularly attractive type of signalling vesicles in atherosclerosis due to the high rate of immune cell migration [83].

TSPANs, the key organizers of migrasomes and exosomes, are extensively expressed in hematopoietic and vascular cells and are involved in both physiological and pathological processes related to thrombosis, hemostasis, angiogenesis, and vascular injuries [17, 84]. TSPAN8 expressed in the membrane of exosomes from cancer cells contributed to a selective recruitment of mRNA and proteins into exosomes, including CD106 and CD49d, which were implicated in exosomes-ECs binding and ECs internalization [85]. Subsequently, the exosomal mRNA and/or proteins induce gene expression that suffices for activation of the quiescent ECs, and these exosomes also allow for the survival of EC progenitors (ECP) [85]. We are unclear whether TSPANs in migrasomes have the same influence on vascular homeostasis, or whether the TSPANs-independent effects of migrasomes have the vascular effects.

Migrasomes were formed in F4/80^+^-microglia/macrophages of ischemic hemispheres of mice that received a standard diet, whereas high salt diet (sodium chloride) enhanced migrasome formation *in vivo*. Sodium chloride can also induce microglial migrasome formation directly *in vitro*, and migrasomes were also detected in postmortem brain tissue of stroke patients [9]. F4/80^+^-migrasomes are co-localized with NeuN, which is expressed in nuclei and cytoplasm of neurons; this suggests that the two different scenarios are possible: migrasomes might carry off fragments of damaged neurons, thus, fulfilling a “cleavage function”; or migrasomes might incorporate the cytosol of intact neurons, thereby, inducing neuronal death and aggravating ischemic cell damage [9]. It is urgent to know whether migrasome is the consequence or the contributor during ischemic stroke.

### 5.5. Migrasomes: the Sensitive Indicators for Early Podocyte Stress and/or Damage

Podocyte-derived EVs have received much more attention for nephrologists and others [86–95], and the urinary podocyte-derived EVs are associated with renal injury in systemic lupus erythematosus [95], preeclampsia [91], and metabolic syndrome [90]. Podocytes control glomerular permeability; they have a higher capacity of motility than other renal cells and can release exosomes and migrasomes, while renal tubular cells secrete less migrasomes; these released migrasomes can be detected in human and mouse urine [10]. It should be noticed that migrasomes and exosomes released from podocyte possess different protein and miRNA profiles; for example, the migrasomes contain more PIGK, miR-1303, miR-490-5p, miR-548a, miR-611, and miR-661 than exosomes isolated from the same cultured podocytes [10]. However, it remains unknown the physiological or pathophysiological functions of these migrasomal miRNA and proteins in podocytes.

The secretion of migrasomes in podocytes was strongly enhanced by puromycin amino nucleoside (PAN), lipopolysaccharide (LPS), or a high concentration of glucose (HG) *in vitro* [10]. Release of migrasomes from podocytes is dependent on Rac-1 (Ras-related C3 botulinum toxin substrate1, a member of Rho family of small molecular weight guanosine triphosphate (GTP)-binding proteins/GTPases), as that Rac-1 inhibitor (EHT 1864) dose dependently inhibited LPS-, PAN-, or HG-induced migrasome formation [10, 96–98]. Urinary migrasomes released from podocyte may serve as a more sensitive indicator than proteinuria for podocyte injury, because that increased urinary migrasome number was detected earlier than elevated proteinuria during PAN-induced nephropathy in mice [10, 99]. Furthermore, it is interesting to know whether the migrasomes in urine have the indicative roles for other renal disorders.

### 5.6. Migrasomes Modulate Cell Proliferation *via* Lateral Transfer of mRNA and Protein

The intact migrasomes can be engulfed by the surrounding cells; what will happen to the recipient cells after the lateral transfer of cellular contents by migrasomes? In addition to protein, migrasomes also enrich in mRNA [2]. So, when migrasomal proteins and mRNA enter the recipient cells with migrasomes, do the laterally transferred mRNA and proteins have any functional consequence in the recipient cells?

Yu's team chose tumor suppressor *PTEN* (phosphatase and tensin homolog on chromosome 10) as an example, as it was among the most abundant group of mRNAs in migrasomes from L929 cells [2]. *PTEN* was firstly identified as a putative protein tyrosine phosphatase gene mutated in human brain, breast, and prostate cancer in 1997 [100]. The SUMO1 modification of PTEN modulates tumorigenesis by controlling its association with the plasma membrane; subsequently, PTEN dephosphorylates and converts phosphatidylinositol 3,4,5-triphosphate (PIP3) into phosphtidylinositol 4,5-biphosphate (PIP2) and, thus, antagonizes the phosphatidylinositol-3-kinase (PI3K)/Akt signalling pathway [101–104]. Human glioblastoma cell line (U87-MG), breast cancer cell line (MDA-MD-468), and prostate cancer cell line (PC3) fail to express PTEN due to frameshift mutations [2, 105–108]. MDA-MB-468 cells incubated with migrasomes from *Pten* knockout L929 cells displayed no *Pten* protein expression with normal p-Akt levels, while U87-MG, MDA-MD-468, and PC3 cells incubated with purified migrasomes from L929 cells; *Pten* protein was expressed; and Akt phosphorylation was dramatically lowered [2]. Furthermore, migrasomes can induce *Pten* protein expression and reduce the phosphorylation of Akt in recipient cell in dose- and time-dependent manners [2]. Both *Pten* protein and mRNA in migrasomes can transfer into the recipient cells, and *Pten* protein modulates Akt phosphorylation in the recipient cell at earlier time points, while *Pten* mRNA plays a more important role at later time points; in which time, it can translate into *Pten* protein [2]. Migrasome-mediated transfer of *Pten* mRNA and Pten protein can inhibit the proliferation of Pten-deficient MDA-MB-468 cancer cells [2], while the effects of migrasome-mediated cargo transfer in U87-MG and PC3 cells are not clear. In addition, migrasomes are also present in multiple cancer cell types [1], while the functions of migrasomes in these cancers and their roles in tumor metastasis *in vivo* remain to be answered (Figure 1).

## 6. Discussion

Migrasome is a novel vesicular structure discovered in migrating cells: during cell migration, projections named “retraction fibers” are pulled from the rear end of cells, and large vesicular structures named “migrasomes” grow on the retraction fibers; when the cell migrates away, the retraction fibers break, and migrasomes are left behind [1] (Figure 1). Migrasomes contain many small vesicles, proteins, mRNA, miRNA, and the damaged mitochondria with low MMP and high ROS [2, 6, 10]. The transporting mechanisms of damaged mitochondria to migrasomes are relatively clear [6]. Nevertheless, the origin of small vesicles inside migrasomes and the sorting and transporting mechanisms of these small vesicles, nucleic acids, and proteins into migrasomes remain to be identified [1, 4, 67, 109].

Once detached from cells, migrasomes can be directly taken up by the surrounding cells and transfer their contents into the surrounding cells (Figure 1); according to these, the physiological and pathophysiological functions of migrasomes partially depend on their interaction with the recipient cells [2, 109], although the mechanisms of this interaction and transfer are unclear. It should be considered that the exchange of information between migrasomes and other membranous organelles, such as exosomes, might also influence the functions of migrasomes [110]. There exist essential interorgan communications from the philosophy of traditional Chinese Medicine based on the record in *Huang-Di-Nei-Jing* (also known as “The Yellow Emperor's Canon of Medicine”) and in “five-viscus (also known as “five-zang” or “five-organ”) correlation theory”, and from the philosophy of Western medicine based on modern anatomy, physiology, molecular genetics, and immunology [111]. Similar to other kinds of EVs, migrasomes have a single layer of membrane (phospholipid bilayer structure), which can protect the contents carried by them from being damaged by digestive enzymes in the environment, while their surface have specific adhesion molecules, which can guide them to the correct recipient cells [1, 112]. Migrasomes exist in urine and blood *in vivo* [6, 10, 19], and they have relative long lifespan [1]; there is no doubt that they can travel into the remote organs *via* blood circulation [113]. Considered that migrasomes can be engulfed by the recipient cells or rupture to release their contents into the environment [1–3], hence, they might act as the essential modulators of interorgan communication *in vivo*.

Migrasomes derived from cells can also rupture and release their luminal contents into the environment in a process named “migracytosis” [1–3, 6, 41] (Figure 1). In zebrafish, it is possible that migrasomes have been generated elsewhere by migrating cells, and after breaking from the retraction fibers, they were “washed” to the embryonic shield cavity by moving cells, thus coordinating organ morphogenesis [3]; yet, these still need further investigation. Migrating cells expel dysfunctional mitochondria by releasing migrasomes (a process referred as “mitocytosisis”) to protect cells against mitochondrial stressor-induced oxidative damage and maintain mitochondrial homeostasis [6, 114–117]. It is interesting to know what will happen to the damaged mitochondria once transporting into migrasomes and leaving behind the migrating cells. It remains unknown whether mitosomes contain normal mitochondria, and whether mitosomes can be engulfed by the surrounding cells, thus transferring mitochondrial information between cells [6]. Mitochondrial ROS is a key regulator of ECM-degrading metalloproteinases transcription and activation [118]; the pairing of integrins with ECM partner protein is essential for the formation of migrasomes [4], which will release into the ECM after formation [1, 119]; and the released migrasomes can discard the damaged mitochondria with high ROS *via* the process of mitocytosis [6]; hence, the influences of mitocytosis or migrasomes on the status of ECM should also be taken into consideration.

Cell migration is the basic phenomena and fundamental mechanisms of modulating body homeostasis and involved in some human diseases, for example, embryogenesis, wound healing, immune defense, cardio-cerebrovascular diseases, eye diseases, cancer biology, osteoporosis, and the chronic inflammatory diseases, e.g., rheumatoid arthritis and multiple sclerosis [17, 22, 23, 120]. Migrasomes are intrinsically associated with cell migration [6]. Until now, the physiological and pathological functions of migrasomes or its related events have been investigated in zebrafish development model, in mild mitochondrial stresses model of neutrophil and macrophages in mice, in human platelets after internalizing SARS-CoV-2, in ischemic stroke mice model, in PAN-induced nephropathy in mice, and in cancer cell proliferation *in vitro* [2, 3, 7, 9, 10] (Figure 1). Among these diseases above, the contents in migrasomes released from the platelets after SARS-CoV-2 infection have not been determined [7]. Considering the universality of migration in modulating homeostasis and diseases, we speculate that the functions of migrasomes are far more than these examined above. More basic and clinical investigations are needed in the future; for example, the investigation on regulatory mechanisms of migrasome biogenesis, release, uptake, and rupture will help us to further understand the function of these charming vesicles.
